# Structural basis of phosphorylation-induced activation of the response regulator VbrR: Activation mechanism of the response regulator VbrR

**DOI:** 10.3724/abbs.2022200

**Published:** 2023-01-09

**Authors:** Sen Hong, Jiaxin Guo, Xue Zhang, Xiaohui Zhou, Peng Zhang, Fang Yu

**Affiliations:** 1 National Key Laboratory of Plant Molecular Genetics Center for Excellence in Molecular Plant Sciences Institute of Plant Physiology and Ecology Chinese Academy of Sciences Shanghai 200032 China; 2 Shanghai Key Laboratory of Plant Molecular Sciences College of Life Sciences Shanghai Normal University Shanghai 200233 China; 3 University of Chinese Academy of Sciences Beijing 100049 China; 4 Department of Pathobiology & Veterinary Science The University of Connecticut Storrs CT 06269 USA.

**Keywords:** two component system, response regulator, crystal structure, DNA binding domain, phosphorylation, activation

## Abstract

Two-component systems typically consist of a paired histidine kinase and response regulator and couple environmental changes to adaptive responses. The response regulator VbrR from *Vibrio parahaemolyticus*, a member of the OmpR/PhoB family, regulates virulence and antibiotic resistance genes. The activation mechanism of VbrR remains unclear. Here, we report the crystal structures of full-length VbrR in complex with DNA in the active conformation and the N-terminal receiver domain (RD) and the C-terminal DNA-binding domain (DBD) in both active and inactive conformations. Structural and biochemical analyses suggest that unphosphorylated VbrR adopts mainly as inactive dimers through the DBD at the autoinhibitory state. The RD undergoes a monomer-to-dimer transition upon phosphorylation, which further induces the transition of DBD from an autoinhibitory dimer to an active dimer and enables its binding with target DNA. Our study suggests a new model for phosphorylation-induced activation of response regulators and sheds light on the pathogenesis of *V*. *parahaemolyticus*.

## Introduction

Two-component systems (TCSs) enable organisms to sense and respond to environmental changes [ 1– 3] . TCSs are involved in the regulation of many cellular activities, including metabolism, motility, quorum sensing, virulence, and antibiotic resistance [ 4– 7] . Canonical TCSs consist of a histidine kinase (HK) and a response regulator (RR). The process of signal transduction involves the transfer of a phosphoryl group from an HK to a cognate RR [8], by which the information of environmental changes is transferred to the cytoplasm. TCSs widely exist in bacteria, archaea, lower eukaryotes, and plants, except for metazoans [ 9, 10] . The absence of TCSs in humans has fueled the design of new drugs targeting them.

RRs typically harbor a receiver domain that contains a highly conserved phosphor-acceptor aspartate and an effector domain that interacts with DNA or protein targets [1]. The receiver domain of the response regulator exists in an equilibrium between the inactive and active states in solution. The structural difference between the two states involves the reorientation of the switch residues (Thr/Ser and Tyr/Phe) within the RD [11]. The phosphoryl-mimic beryllium fluoride (BeF _3_^–^) and a D to E point mutation are the two most common methods used to study activated RRs [ 11, 12] . Phosphorylation induces reorientation of the switch residues in RD and hence dimerization of RD [11].

Three complex structures of full-length active OmpR/PhoB response regulator family members, including KdpE [13], PmrA [14], and PhoP [15], bound to target DNA have been reported, showing that the active RD and DBD are homodimers with different RD-DBD interfaces. In addition, a number of full-length structures of inactive OmpR/PhoB subfamily members, including DrrD [16]and DrrB [17] from *Thermotoga maritima*, PrrA [18], MtrA [19], and RegX3 [20] from *Mycobacterium tuberculosis*, have been reported in the monomeric state. Among these, the RDs of PrrA, MtrA, DrrB, and RegX3 interact with DBDs through their dimerization interface α4-β5-α5, suggesting a potential mechanism of how these proteins remain inactivated in solution. However, DrrD remains inactivated even though its RD and DBD have no interactions. Therefore, response regulators may use different mechanisms for their inhibition and activation.

*Vibrio parahaemolyticus* is an important gram-negative bacterial pathogen associated with marine organisms and the leading cause of seafood-associated gastroenteritis in humans [ 21– 23] . The VbrK/VbrR TCS of *V*. *parahaemolyticus* consists of a histidine kinase VbrK and its cognate response regulator VbrR. The VbrK/VbrR TCS is activated by host-derived nitrite during infection, which leads to the downregulation of Type-3 secretion system-1 (T3SS1) via repression of its positive regulator *exsC* for maximal virulence [ 24– 26] . VbrR is a member of the OmpR/PhoB family and contains an N-terminal receiver domain (RD) and a C-terminal DNA-binding domain (DBD), connected by a flexible linker, and can directly bind with the promoter region of *exsC* to mediate its downregulation [24]. Here, we report the crystal structures of RD (both free and BeF _3_^–^ bound), the DBD, and the full-length VbrR-DNA complex. Our results illustrate a new molecular mechanism underlying phosphorylation-induced activation of the VbrR protein.

## Materials and Methods

### Gene cloning and protein purification

The gene encoding VbrR was amplified by PCR from *Vibrio parahaemolyticus* genomic DNA. Fragments encoding full-length VbrR, receiver domain (RD, 1–117), and DNA-binding domain (DBD, 125–220) were inserted into the first multiple-cloning site of the pRSFDuet-1 vector with a 6× His tag at the N-terminus. Site-directed mutagenesis of VbrR was performed by one-step PCR; all mutants were validated by DNA sequencing. All plasmids were expressed using *E*. *coli* BL21 (DE3) strains in LB medium. Protein expression was induced with 0.25 mM IPTG at OD _600_ around 0.8 after approximately 12 h at 37°C. Cells were collected by centrifugation and homogenized in buffer A (20 mM Tris-HCl pH 8.0, 100 mM NaCl) supplemented with 1 mM PMSF. Cells were lysed using a French Press at 15000 psi. Cell debris was removed by centrifugation. The supernatant was loaded onto a Ni-NTA column preequilibrated with buffer A, washed with buffer A, and eluted with buffer A plus 250 mM imidazole, and further purified by size-exclusion chromatography using a Superdex 200 column (Cytiva, Wilmington, USA) preequilibrated with buffer A. Peak fractions containing target proteins were collected and concentrated to approximately 10 mg/mL for crystallization. For the purification of activated receiver domain [11], 5.3 mM BeSO _4_·4H _2_O, 35 mM NaF, and 7 mM MgCl _2_ were added to buffer A. For the binding assays using biolayer interferometry (BLI), purified proteins were prepared, except that buffer A was replaced by PBS buffer (10 mM phosphate buffer pH 7.4, 2.7 mM KCl, 137 mM NaCl). Selenomethionine (SeMet)-labelled protein expression was performed as described previously [27].

### Size-exclusion chromatography coupled multiangle laser light scattering (SEC-MALLS)

A Superdex-200 10/30 column was used for SEC-MALLS and preequilibrated with buffer A, which was filtered through a 0.22 μM filter and sonicated. The system was equilibrated with buffer A overnight at 0.5 mL/min. Protein solution (100 μL, ~10 mg/mL) was injected, and the data were recorded. Data were processed using the ASTRA software package version 5.4.2.10 (Wyatt Technology, Santa Barbara, USA). For activated receiver domain detection, buffer A was supplemented with 5.3 mM BeSO _4_·4H _2_O, 35 mM NaF, and 7 mM MgCl _2._ The curves for UV280, differential refractive index, and light scattering were processed using Origin software (OriginLab, Northampton, USA).

### Binding assays using biolayer interferometry

The *K*_D_ values for VbrR-DNA interactions were determined using an Octet RED system (Sartorius, Gottingen, Germany) in 96-well microplates at 25°C. The protein was biotinylated using a kit (Bomeida, Suzhou, China) according to the manufacturer’s instructions. Biotinylated proteins were diluted to 50 μg/mL in PBS buffer. Proteins were immobilized onto streptavidin biosensors (Sartorius) and incubated with DNA prepared from synthetic oligonucleotides by an annealing procedure (95°C for 5 minfollowed by cooling in 2°C steps to reach 16°C) to form double-stranded DNA in PBS buffer. Concentrations of DNA fragments were optimized for all experiments. Signals for layer thickness (nm) were recorded in real time, with *K*_D_ values calculated by fitting to a 1:1 binding model using Data Analysis Software v7.1 (Sartorius). Association and dissociation curves were drawn using Origin software. All experiments were repeated at least twice.

### Crystallization, data collection, and structure determination

For crystallization screens, a series of lengths of DNA, with either blunt ends or overhangs of one or two bases, were mixed with monomeric VbrR at a 1.2:1 molar ratio and incubated overnight at 4°C. VbrR -26 bp DNA crystals were grown at 20°C using the sitting-drop vapour diffusion method by mixing 0.4 μL of the protein‒DNA mixture with 0.4 μL of reservoir solution containing 0.2 M MES pH 6.5, and 10% (W/V) PEG4000. However, only poor diffraction (~8 Å) was initially achieved. Therefore, we used postcrystallization treatment [28] to improve the diffraction quality. A condition containing 0.2 M NaCl, 0.1 M MES, pH 6.6, and 20% PEG4000 was used as a dehydrating agent for a week; crystal diffraction was improved to ~4.6 Å by this method. Crystals of both native and SeMet-labelled VbrR were grown at 20°C using the sitting-drop vapour diffusion method by mixing 0.4 μL of protein with 0.4 μL of reservoir solution containing 0.2 M NaCl, 0.1 M HEPES, pH 7.0, and 20% (W/V) PEG6000. RD-BeF _3_^–^ crystals were obtained at 20°C using a reservoir solution containing 0.1 M CH _3_COONa·3H _2_O, pH 4.5, and 25% (W/V) PEG3350. Crystals were directly flash-frozen in liquid nitrogen prior to data collection and supplemented with 25% glycerol at 100 K. Data from VbrR-DNA crystals were collected at the Shanghai Synchrotron Radiation Facility (SSRF, Shanghai, China) beamline BL17U and were processed using HKL3000 [29]. Native VbrR, SeMet-labelled VbrR, and RD-BeF _3_^–^ crystals were collected at the SSRF beamline BL19U1. The SeMet-labelled VbrR structure was solved using the single-wavelength anomalous dispersion (SAD) method. Selenium sites were solved, and initial phases were calculated using the HKL3000 package. However, the crystal structure of VbrR only contained a homodimer of the DBD. The structure of RD-BeF _3_^–^ was solved by molecular replacement using Phenix [30], and the structure of the receiver domain of KdpE (PDB ID: 4KFC) [20] was used as the search model. The structure of the VbrR-DNA complex was solved by molecular replacement using the structures of the RD and DBD-DNA structure of KdpE (PDB ID: 4KFC) as the initial search model. All structure models were refined with Phenix and manually built using Coot [31]. Data collection and model refinement statistics are shown in Supplementary Table S1. Graphics were drawn using PyMOL (Schrodinger LLC, New York, USA).

### Analysis of mutant DNA of the protein-DNA interaction by biolayer interferometry

VbrR protein was prepared for the binding assay in PBS buffer and immobilized onto streptavidin biosensors (Sartorius). A series of mutant DNA fragments were prepared from synthetic oligonucleotides by an annealing procedure to form double-stranded DNA molecules in PBS buffer, as described above. VbrR-coated sensors were then incubated with different mutant DNA fragments at 80 μM for 30 s to record the signal resulted from association and then incubated with PBS buffer for 30 s to record the signal resulted from dissociation. The association and dissociation curves were drawn using Origin software.

## Results

### Characterization of VbrR protein

**Figure 1:**
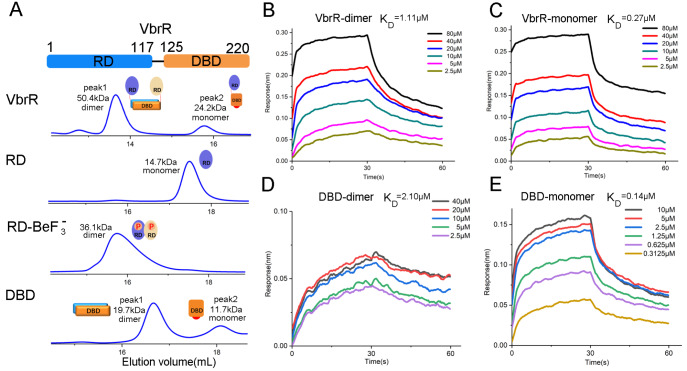
Figure 1 Solution state of VbrR and its binding affinity for DNA (A) Schematic view of VbrR and size exclusion chromatography profiles of VbrR, receiver domain (RD), receiver domain bound to BeF 3 – (RD-BeF 3 –), and DNA-binding domain (DBD). Molecular weights were determined by SEC-MALLS. (B‒E) BLI analysis of the interaction of DNA with the (B) VbrR dimer, (C) VbrR monomer, (D) DBD dimer, and (E) DBD monomer.

To study the behavior of VbrR protein in solution, we expressed and purified recombinant full-length VbrR (VbrR), as well as its receiver domain (RD, 1–117) and DNA-binding domain (DBD, 125–220), and carried out the evaluation using SEC-MALLS ( Figure 1A and Supplementary Figure S1). Both VbrR and DBD exist mainly as dimers (peak 1) and a small amount as monomers (peak 2) in solution. Whereas the RD exists almost exclusively as monomers, and the addition of BeF _3_^–^ induces a monomer to dimer transition in solution. However, full-length VbrR underwent precipitation when BeF _3_^–^ was added to the protein sample ( Supplementary Movie S1).

To test the DNA binding ability of the purified proteins in various states, we measured their individual dissociation constant ( *K*_D_) with the 49 bp *exsC  *promoter DNA [24] using the BLI method. The results show that VbrR monomer binds with DNA much stronger than the dimer, with *K*_D_ of 0.27 μM and 1.11 μM, respectively ( Figure 1B,C). The binding affinity of DBD monomer for DNA is also much stronger than that of DBD dimer, with *K*_D_ of 0.14 μM and 2.10 μM, respectively ( Figure 1D,E). In summary, the DNA binding assay suggests that monomeric VbrR or DBD binds with DNA more tightly, which implies that the native dimer in solution may represent an autoinhibitory state.

### Structure of the VbrR-DNA complex

**Figure 2:**
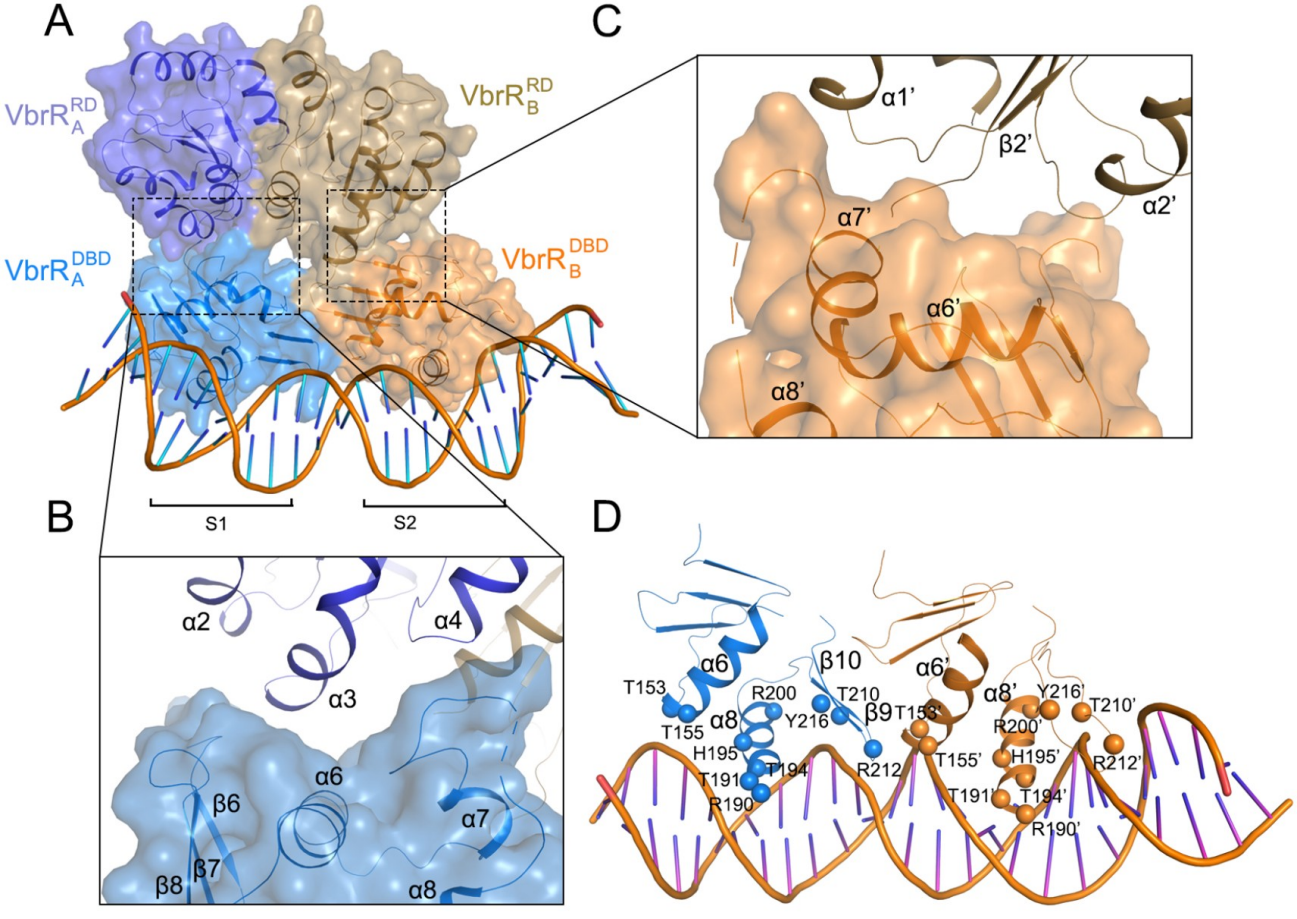
Figure 2 Overall structure of the VbrR-DNA complex (A) Surface representation of the VbrR-DNA complex. RDs of chains VbrR A and VbrR B are shown in purple and beige, respectively, whereas the DBDs of chains VbrR A and VbrR B are shown in blue and orange, respectively. DNA is shown as a cartoon. S1 and S2 are the half-sites. (B,C) Zoomed-in views of the RD-DBD interfaces of (B) VbrR A and (C) VbrR B. (D) Zoomed-in views of DBDs bound to DNA. The key residues are shown as spheres. For clarity, α7 is omitted.

To identify DNA fragments suitable for crystallization, various lengths of DNA fragments within the 49 bp *exsC* promoter segment were assessed using BLI. A 30 bp DNA fragment was selected because it has similar binding affinity compared to the 49 bp DNA segment, with dissociation constants of 0.69 μM and 0.77 μM, respectively ( Supplementary Figure S2). Therefore, the promoter sequence was narrowed down to a 30 bp fragment. To gain insights into the DNA binding mode of VbrR, we cocrystallized the monomeric VbrR and DNA fragments of various lengths within the 30 bp fragment, with either blunting ends or overhangs of one or two bases. Crystals of VbrR in complex with a blunt-ended 26 bp DNA fragment were obtained, and the resulting structure of the VbrR-DNA complex was determined to 4.6 Å resolution to allow for the denotation of the secondary structure elements of the RD and DBD domains ( Figure 2A and Supplementary Table S1). Eight molecules, corresponding to four VbrR dimers and two DNA fragments, were modelled in the asymmetric unit ( Supplementary Figure S3). Two dimers (complex-1 and complex-3) bind with DNA fragments in the forward direction, while the other two (complex-2 and complex-4) bind with DNA fragments in the reverse direction, which may be due to crystal packing. The four VbrR dimers adopt very similar conformation, with RMSD values of 0.73–0.80 Å for 215 C _α_ atoms.

We focused on complex-1, which represents the active conformation of VbrR dimer binding with *exsC* promoter DNA, to analyze structural details. In the VbrR-DNA structure, each VbrR protomer contains one RD and one DBD, connected by a disordered linker. The two RDs form a symmetric head-to-head dimer using the α _4_-β _5_-α _5_ interface, and the two DBDs form a head-to-tail dimer and bind with DNA half-sites S1 (TTCTAAT) and S2 (TTCATCG), respectively ( Figure 2A). It is noteworthy that the two promoters in the complex adopt different conformations, which is attributed to the relative orientation of the RDs and DBDs. Specifically, the RD and DBD interaction surfaces in protomers A and B are different ( Figure 2B,C). In protomer A, the interaction surface involves α2-α4 from the RD domain and β7-β8 loop, α6, and α7-α8 loop from the DBD domain, whereas it involves α1’, α1’-β2’ loop, and α2’ from the RD domain and α6’, α7’, α7’-α8’ loop from the DBD domain in protomer B.

The DBD from each protomer uses the same structural elements for DNA binding. Each DBD places its recognition helix-α8/α8’ into the DNA major groove, where it recognizes 7 bp of promoter DNA, and the C-terminus of α6/α6’ also involves DNA binding. In addition, the β-hairpin connecting β9-β10/β9’-β10’ dips into the DNA minor groove ( Figure 2D). However, the side chain conformation of the structural elements is ambiguous due to the modest resolution of the structure. To confirm the binding mechanism, we constructed the following mutants: T153A and T155A within the α6 helix; R190A, T191A, T194A, H195A, and R200A within the α8 helix; and T210A, R212A, and Y216A within the β-hairpin. The DNA binding affinity of each mutant was evaluated using BLI. The results show that the R190A mutant completely abolishes DNA binding ( Table 1), consistent with the location of this residue in the recognition helix. The DNA binding affinity of the T155A and T210A mutants is significantly reduced, while that of the T153A, T191A, H195A, R200A, R212A, and Y216A mutants is moderately reduced, suggesting that these residues play certain roles in DNA binding. To further validate whether the DNA two half sites are equally important for VbrR binding, we tested the binding capacity of DNA harboring mutations within the *exsC*promoter sequence. Mutation of dinucleotides within this promoter decreases affinity, as evidenced by BLI analysis ( Supplementary Figure S4A‒C). Specifically, the M3 mutant exhibited the least binding ability relative to wild-type DNA ( Supplementary Figure S4A,B), and mutation of dinucleotides within DNA half-site S1 led to a more significant reduction in affinity than did half-site S2, suggesting that half-site S1 could be more important in DNA binding.

**Table:** **Table 1** DNA binding affinity of wild-type and mutant VbrR

VbrR	Dissociation constant/ *K*_D_ (μM)*
WT	0.77±0.06
T153A	4.27±0.26
T155A	12.61±0.90
R190A	No binding
T191A	2.68±0.15
T194A	0.51±0.02
H195A	3.32±0.25
R200A	3.03±0.24
T210A	12.30±1.04
R212A	4.64±0.32
Y216A	2.48±0.19

*The binding affinity of DNA to wild-type and mutant VbrR is assayed by BLI. Data are presented as the mean±SD.

Taken together, these data strongly suggest that the VbrR-DNA complex structure is in the active conformation. To study the activation mechanism of VbrR, numerous efforts have been made to obtain the full-length inactive conformation of the VbrR structure but have failed. Instead, we managed to determine the structures of the RD and DBD in the inactive conformation.

### RD structure in the active and inactive conformations

**Figure 3:**
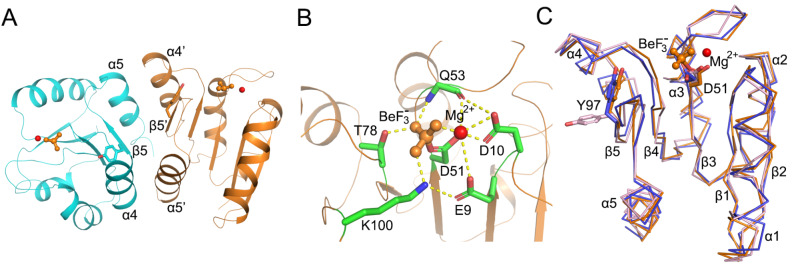
Figure 3 The RD of VbrR in different states (A) The active RD dimer (protomers A and B) with monomers shown in orange and cyan, respectively. (B) Zoomed-in views of active sites showing coordinating residues. BeF 3 – (orange) is shown as sticks. Mg 2+is shown as a sphere (red). (C) Structural alignment of the RD in the presence of BeF 3 – (protomer A, orange), the RD in the absence of BeF 3 – (protomer L, pink), and the RD within the VbrR-DNA complex (VbrR A RD, purple). The switch residue Tyr97 is shown as sticks.

We purified and crystallized the RD protein in the presence of BeF _3_^–^ and solved the corresponding structure to 2.80 Å ( Supplementary Table S1). The asymmetric unit contains fourteen RD molecules, comprising four dimers (AB, CD, EF, and GH) and six monomers (I to N). However, the BeF _3_^–^ molecule only binds to molecules A, B and E, among which molecules A and B form a homodimer. The AB homodimer superimposes very well with the RD domain structure within the VbrR-DNA complex (RMSD is 0.92 Å), and the dimer interface is composed of α4-β5-α5 from both protomers, both of which represent an active dimer conformation ( Figure 3A and Supplementary Figure S5). The homodimer interaction surface is maintained mainly by four intermolecular salt bridges formed between residues Asp91, Asp96, Arg110 and Arg112 from each protomer, and all these residues are highly conserved among members of the OmpR/PhoB subfamily ( Supplementary Figures S6 and S7). The other three dimers (CD, EF, and GH) in the asymmetric unit also adopt an active conformation similar to that of AB, even without BeF _3_^–^ binding, probably due to crystal packing or high protein concentration. Similar observations have been reported in other RD structures [11].

The BeF _3_^–^ molecule is noncovalently bound to the active site Asp51, where it occupies four coordination sites with residues Thr78, Lys100, Gln53, and Mg ^2+^ ( Figure 3B). Mg ^2+^ forms five coordinates with BeF _3_^–^, residues Glu9, Asp10, and Gln53. Residues with side chains involved in the formation of coordinates with BeF _3_^–^ and Mg ^2+^ are also conserved among members of the OmpR/PhoB family ( Supplementary Figure S7).

Interestingly, six molecules (I to N) exist as monomers in the asymmetric unit. Structural comparison of monomer L with protomer A in the active dimers reveals significant conformational difference at the dimer interface. In particular, the residue Tyr97 adopting an inward-facing conformation in the active dimer changes to an outward-facing conformation in all the monomers ( Figure 3C). Such a conformational change could turn RD dimerization on/off and has been considered a switch between the active state and inactive state of RD [11].

### DBD structure in the autoinhibitory conformation

**Figure 4:**
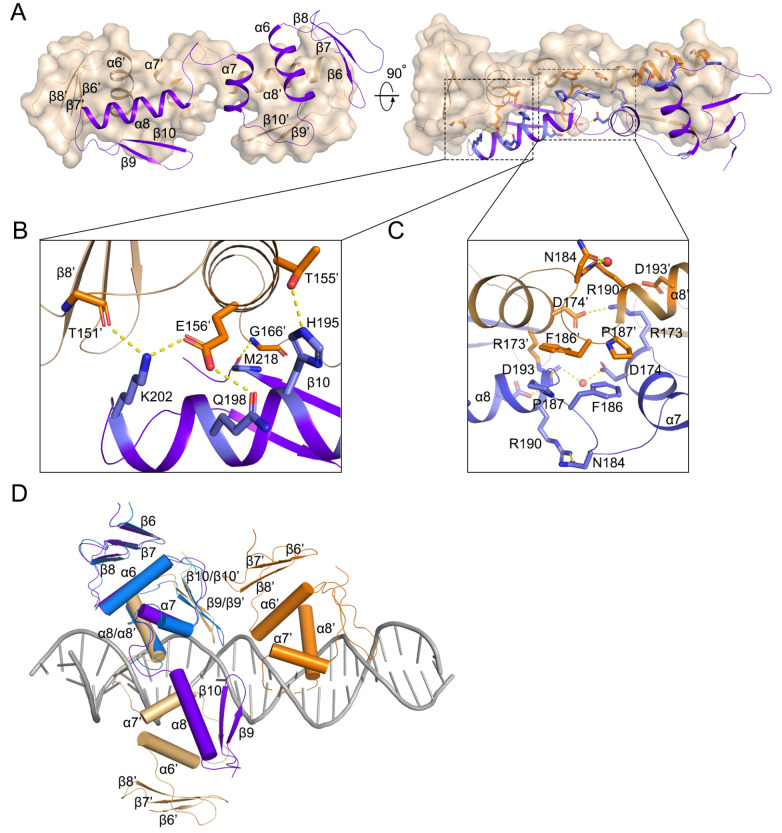
Figure 4 Conformations of DBDs (A) DBD dimer with monomers shown in purple and beige, respectively. (B,C) Zoom-in views of the interactions of the α6-α7-α8 interface. (D) Structural alignment of the DBD structure with DBD domains within the VbrR-DNA complex structure. DNA is shown as sticks (gray).

To gain insights into the DNA-binding-induced conformational changes of the DBD, we purified the full-length VbrR protein and used it for crystallization, but the crystals obtained only contained the DBD. Finally, the DBD structure was determined to 1.45 Å resolution using the SAD method. The structure of DBD forms a centrosymmetrical homodimer ( Figure 4A). Each DBD protomer contains five β-strands (β6-β10/β6’-β10’) and three helixes (α6-α8/α6’-α8’). The DBD homodimer interface involves α6-α8 from each protomer, and extensive hydrophilic and hydrophobic interactions are identified ( Figure 4B,C). Specifically, residues Asp174, Asp193, His195, Gln198, and Lys202 from one protomer form hydrogen bonds with residues Thr151, Thr155, Glu156, and Arg173 from the neighboring protomer. Residues Phe186 and Pro187 from each protomer stack against each other to further stabilize the homodimer.

In contrast to the DBD in the VbrR-DNA complex structure, which forms a compact and globular conformation, the separated DBD structure adopts an extended conformation with dimensions of 31.8 Å ×36.6 Å×72.2 Å ( Figure 4A,D). It is worth noting that the DNA binding motifs α6, α8 and β9-β10 hairpin of DBD in the VbrR-DNA complex structure are blocked in the separated DBD dimer structure. The key residue Arg190 from recognition helix α8 forms two hydrogen bonds with residue Asn184 ( Figure 4C). The structure data are consistent with the biochemical results, suggesting that the DBD dimer binds with promoter DNA much more weakly than the monomer ( Figure 1D,E). Therefore, it is conceivable that the DBD in solution intends to form an autoinhibitory dimer conformation that precludes DNA binding.

## Discussion

It is well known that the phosphorylation of the receiver domain of response regulators induces conformational changes, which can activate the DNA binding affinity of the DNA binding domain. A number of structures have been reported, suggesting the activation mechanism [ 13– 22] . The overall structure of the VbrR-DNA structure shows significant differences, especially at the RD-DBD interfaces ( Supplementary Figure S8). We observed that VbrR exists mainly as dimers in solution, while RD and DBD exist as monomers and dimers, respectively, in solution. This suggests that the inactivated VbrR dimer is mediated through the DBD. Since RD undergoes a monomer-to-dimer transition upon phosphorylation ( Figure 1A), this raises the question of how the phosphorylation of RD changes the DBD from an autoinhibitory conformation to an active or DNA-binding conformation.

**Figure 5:**
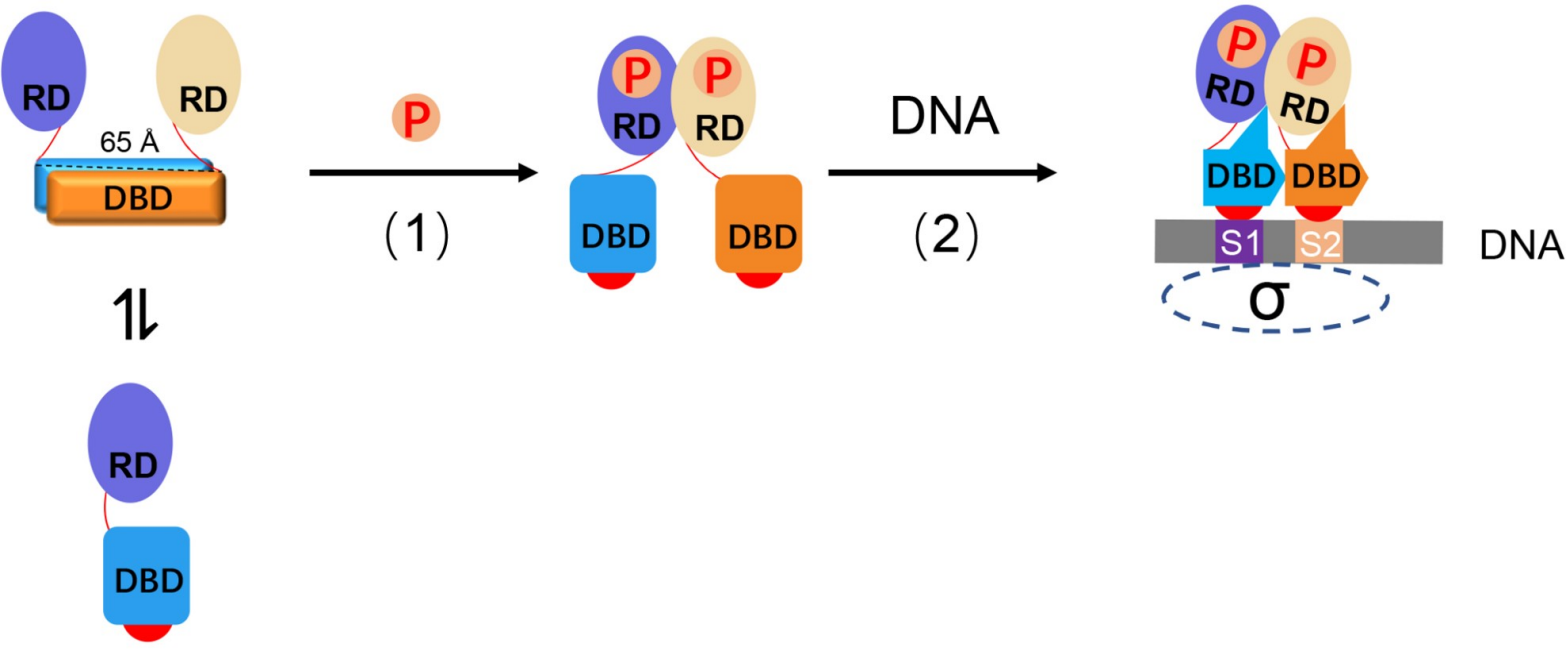
Figure 5 Model for conformational changes induced by phosphorylation to form a stable VbrR-DNA complex (1) Formation of active dimers induced by phosphorylation through α4-β5-α5 interfaces of RDs. (2) Active dimers bind to DNA to form a stable VbrR-DNA complex. The colors used for the RD and DBD domains in the VbrR-DNA complex are the same as those described in Figure 2A. S1 and S2 are the half-sites. The sigma factor is shown as an ellipse of the dotted line.

After further inspection of the structures, we found that the RD connects to the DBD through an 8-residue linker, but this linker is disordered either in the VbrR-DNA complex structure or domain structures. We measured the distance of the N-termini of the two DBDs in the autoinhibitory state, which was approximately 65 Å( Figure 5). However, the maximum length of the main chain of 16 residues is 57 Å. This means that RD dimerization requires a conformational change of DBD, probably from the autoinhibition state to the activation state, and the dimeric DBD activation state binds with target DNA very tightly. Based on these findings, we propose a working model for VbrR, which functions as a repressor of *exsC* transcription ( Figure 5). In the cell or solution, VbrR proteins exist mainly as inhibitory dimers formed through the DBD, and phosphorylation of RDs allows the formation of RD dimers, which in turn changes the conformation of the DBD from an inhibitory state to an active state. Then, the activated VbrR binds DNA through the DBD active dimer to form a stable VbrR-DNA complex via intra- and intermolecular interactions, competing with the binding of the sigma factor to the *exsC* promoter and thereby repressing *exsC* transcription. In conclusion, our work reveals a specific molecular mechanism of VbrR activation, in which the DBD undergoes a large conformational change from autoinhibition to activation ( Supplementary Movie S2).

## Data Availability

The atomic coordinates of the VbrR-DNA, DBD, and RD-BeF _3_^–^structures have been deposited in the Protein Data Bank with accession codes of 7E1B, 7E1F, and 7E1H, respectively.

## Supporting information

147supplementary_data

Moive_S2

Moive_S1

## Supplementary Data

Supplementary data is available at *Acta Biochimica et Biophysica Sinica* online.

## References

[1] Stock AM, Robinson VL, Goudreau PN (2000). Two-component signal transduction. Annu Rev Biochem.

[2] Zschiedrich CP, Keidel V, Szurmant H (2016). Molecular mechanisms of two-component signal transduction. J Mol Biol.

[3] Jacob-Dubuisson F, Mechaly A, Betton JM, Antoine R (2018). Structural insights into the signalling mechanisms of two-component systems. Nat Rev Microbiol.

[4] Gao R, Stock AM (2009). Biological insights from structures of two-component proteins. Annu Rev Microbiol.

[5] Li J, Wang C, Yang G, Sun Z, Guo H, Shao K, Gu Y (2017). Molecular mechanism of environmental
^d^-xylose perception by a XylFII-LytS complex in bacteria. Proc Natl Acad Sci USA.

[6] Groisman EA (2016). Feedback control of two-component regulatory systems. Annu Rev Microbiol.

[7] Neiditch MB, Federle MJ, Miller ST, Bassler BL, Hughson FM (2005). Regulation of LuxPQ receptor activity by the quorum-sensing signal autoinducer-2. Mol Cell.

[8] Casino P, Rubio V, Marina A (2009). Structural insight into partner specificity and phosphoryl transfer in two-component signal transduction. Cell.

[9] Wuichet K, Cantwell BJ, Zhulin IB (2010). Evolution and phyletic distribution of two-component signal transduction systems. Curr Opin Microbiol.

[10] Ortet P, Whitworth DE, Santaella C, Achouak W, Barakat M (2015). P2CS: updates of the prokaryotic two-component systems database. Nucleic Acids Res.

[11] Bachhawat P, Stock AM (2007). Crystal structures of the receiver domain of the response regulator PhoP from
*Escherichia coli* in the absence and presence of the phosphoryl analog beryllofluoride. J Bacteriol.

[12] Lin W, Wang Y, Han X, Zhang Z, Wang C, Wang J, Yang H (2014). Atypical OmpR/PhoB subfamily response regulator GlnR of actinomycetes functions as a homodimer, stabilized by the unphosphorylated conserved Asp-focused charge interactions. J Biol Chem.

[13] Narayanan A, Kumar S, Evrard AN, Paul LN, Yernool DA (2014). An asymmetric heterodomain interface stabilizes a response regulator–DNA complex. Nat Commun.

[14] Lou YC, Weng TH, Li YC, Kao YF, Lin WF, Peng HL, Chou SH (2015). Structure and dynamics of polymyxin-resistance-associated response regulator PmrA in complex with promoter DNA. Nat Commun.

[15] He X, Wang L, Wang S (2016). Structural basis of DNA sequence recognition by the response regulator PhoP in Mycobacterium tuberculosis. Sci Rep.

[16] Buckler DR, Zhou Y, Stock AM (2002). Evidence of intradomain and interdomain flexibility in an OmpR/PhoB homolog from Thermotoga maritima. Structure.

[17] Robinson VL, Wu T, Stock AM (2003). Structural analysis of the domain interface in DrrB, a response regulator of the OmpR/PhoB subfamily. J Bacteriol.

[18] Nowak E, Panjikar S, Konarev P, Svergun DI, Tucker PA (2006). The structural basis of signal transduction for the response regulator PrrA from Mycobacterium tuberculosis. J Biol Chem.

[19] Friedland N, Mack TR, Yu M, Hung LW, Terwilliger TC, Waldo GS, Stock AM (2007). Domain orientation in the inactive response regulator
*Mycobacterium tuberculosis* MtrA provides a barrier to activation. Biochemistry.

[20] King-Scott J, Nowak E, Mylonas E, Panjikar S, Roessle M, Svergun DI, Tucker PA (2007). The structure of a full-length response regulator from Mycobacterium tuberculosis in a stabilized three-dimensional domain-swapped, activated state. J Biol Chem.

[21] Letchumanan V, Chan KG, Lee LH (2014). Vibrio parahaemolyticus: a review on the pathogenesis, prevalence, and advance molecular identification techniques. Front Microbiol.

[22] Su YC, Liu C (2007). Vibrio parahaemolyticus: a concern of seafood safety. Food Microbiol.

[23] Hubbard TP, Chao MC, Abel S, Blondel CJ, Abel zur Wiesch P, Zhou X, Davis BM (2016). Genetic analysis of
*Vibrio parahaemolyticus* intestinal colonization. Proc Natl Acad Sci USA.

[24] Gu D, Zhang Y, Wang Q, Zhou X (2020). S-nitrosylation-mediated activation of a histidine kinase represses the type 3 secretion system and promotes virulence of an enteric pathogen. Nat Commun.

[25] Zhou X, Konkel ME, Call DR (2010). Regulation of type III secretion system 1 gene expression in
*Vibrio parahaemolyticus* is dependent on interactions between ExsA, ExsC, and ExsD. Virulence.

[26] Liu J, Lu SY, Orfe LH, Ren CH, Hu CQ, Call DR, Avillan JJ (2016). ExsE is a negative regulator for T3SS gene expression in vibrio alginolyticus. Front Cell Infect Microbiol.

[27] Qi X, Lin W, Ma M, Wang C, He Y, He N, Gao J (2016). Structural basis of rifampin inactivation by rifampin phosphotransferase. Proc Natl Acad Sci USA.

[28] Heras B, Martin JL (2005). Post-crystallization treatments for improving diffraction quality of protein crystals. Acta Crystlogr D Biol Crystlogr.

[29] Minor W, Cymborowski M, Otwinowski Z, Chruszcz M (2006). *HKL*-3000: the integration of data reduction and structure solution – from diffraction images to an initial model in minutes. Acta Crystlogr D Biol Crystlogr.

[30] Adams PD, Afonine PV, Bunkóczi G, Chen VB, Davis IW, Echols N, Headd JJ (2010). *PHENIX*: a comprehensive Python-based system for macromolecular structure solution. Acta Crystlogr D Biol Crystlogr.

[31] Emsley P, Lohkamp B, Scott WG, Cowtan K (2010). Features and development of
*Coot*. Acta Crystlogr D Biol Crystlogr.

