# Supplementary material for: Structural basis of phosphorylation-induced activation of the response regulator VbrR: Activation mechanism of the response regulator VbrR
Source: Acta Biochim Biophys Sin (Shanghai). 2023 Jan 9;55(1):43–50. doi: 10.3724/abbs.2022200 (PMC10157535; doi:10.3724/abbs.2022200)
Supplement: 147supplementary_data [file 147supplementary_data.pdf]

Supplementary Data

Supplementary Figures

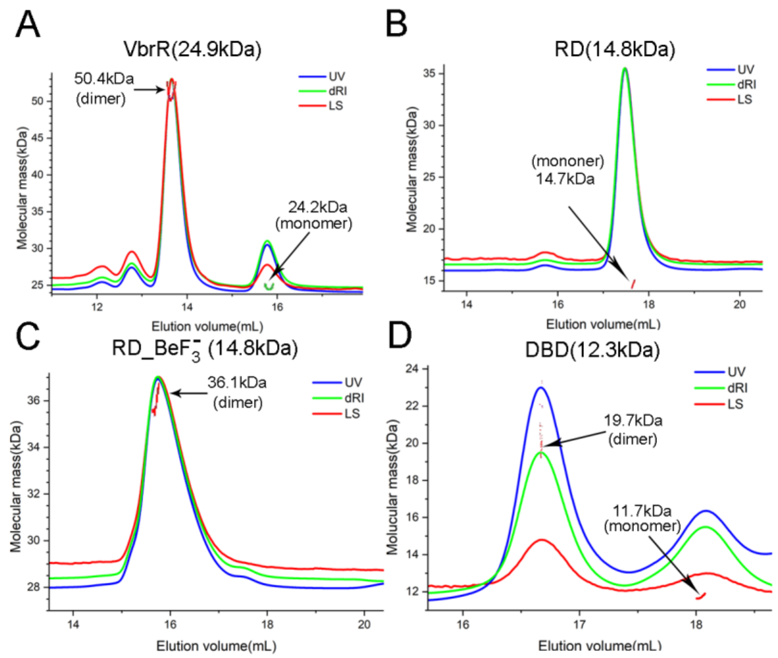

Supplementary Figure S1. Size exclusion chromatography-multi angle laser light scattering (SEC-MALLS) analysis of VbrR in different states (A–D) Representative SEC-MALLS analysis of (A) VbrR, (B) RD in the absence of BeF_3^-, (C) RD in the presence of BeF_3^-, and (D) DBD. Curves show data of absorbance UV280 (UV, blue), differential refractive index (dRI, green), and laser scattering (LS, red). The red/green dotted lines indicate molecular mass calculated of protein samples eluted from a Superdex-200 10/300 column. Theoretical sequence weight of protein is shown on the top.

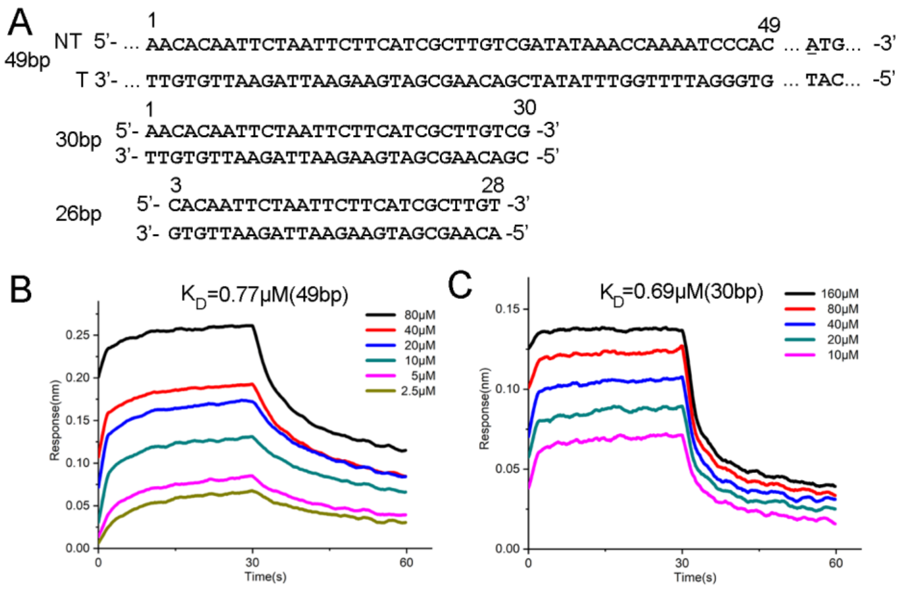

Supplementary Figure S2. Binding affinity of VbrR for promoter DNA (A) The sequences of the double-stranded DNA used in this study. The sequence of a blunt-ended 26 bp DNA segment used for determination of the VbrR-DNA structure is also shown. Binding affinity of VbrR immobilized onto streptavidin-coated biosensors was analyzed by incubating with multiple concentrations of DNA fragments of (B) 49 bp (2.5–80 μM) and (C) 30 bp (10–160 μM).

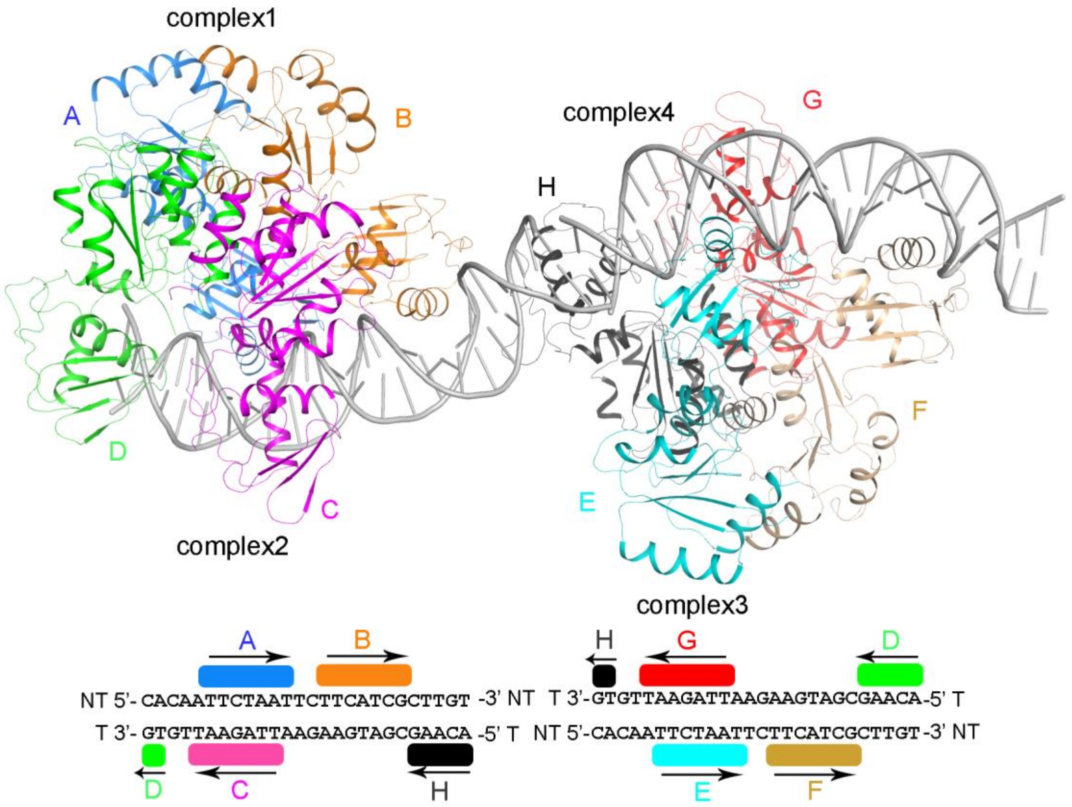

Supplementary Figure S3. VbrR-DNA complex structure in the asymmetric unit

Eight protomers (A to H) are shown in blue, orange, magentas, green, cyan, beige, red, and black, respectively. DNA is shown in gray. The 26bp DNA segment that VbrR recognizes is shown at the bottom. The 7-bp of DNA contacted by each protomer recognition helix is represented by bar of the corresponding color.

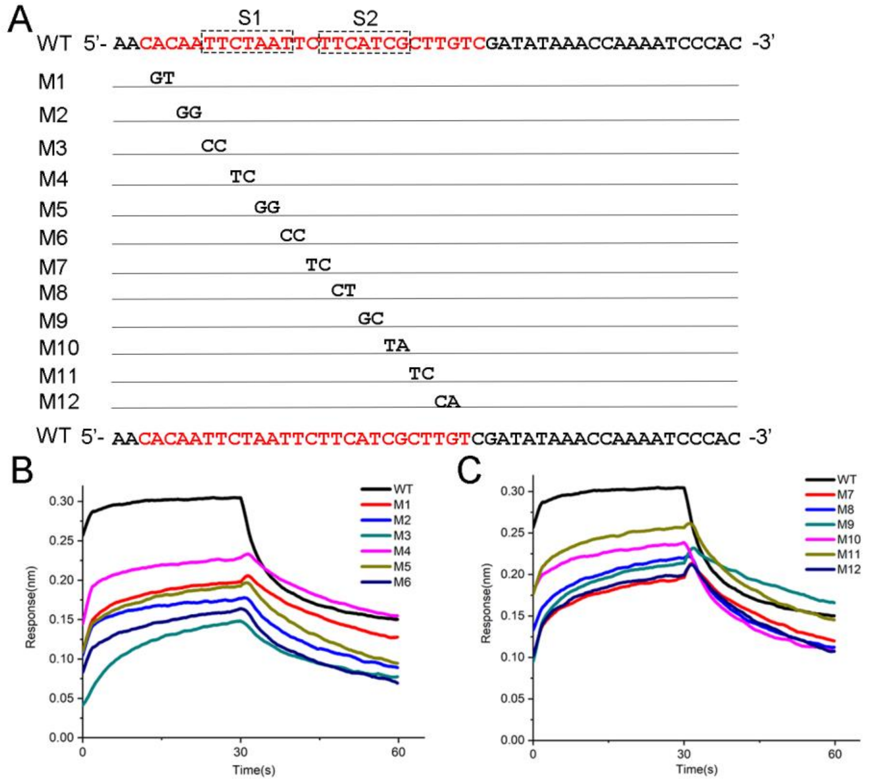

Supplementary Figure S4. VbrR binding to the 49 bp promoter carrying mutation (A) Summary of BLI data using the 49 bp DNA segment and modified oligonucleotides (only specific two substitutions are noted) are presented. WT promoter sequence is shown on the top and at the bottom, with the 26 bp DNA segment that VbrR binds shown in red. The 7 bp of DNA contacted by VbrR recognition helix is shown in black dotted line box. (B,C) BLI analysis of the interactions of VbrR with (B) M1-M6 mutants and (C) M7-M12 mutants.

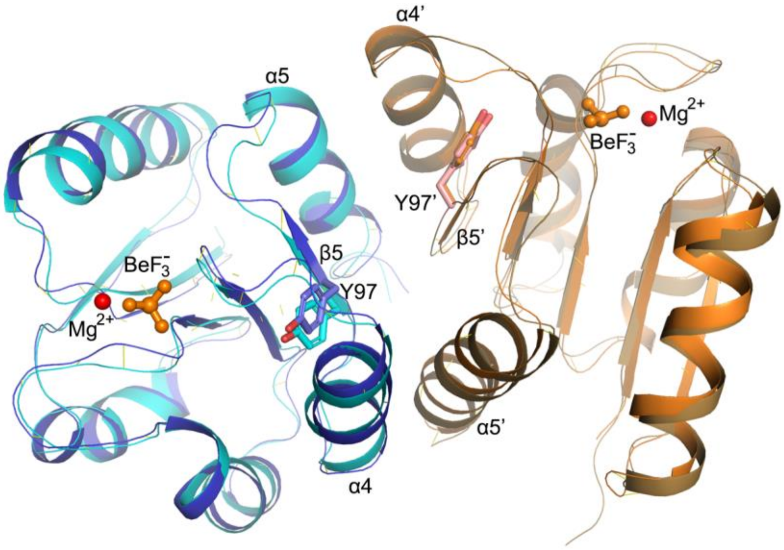

Supplementary Figure S5. Structural alignment of the active RD dimer (protomer A and B) and RD domains within the VbrR-DNA complex RDs of protomer A and B are shown in cyan and orange, respectively, whereas the RD domains within the VbrR-DNA complex are shown in blue and beige, respectively. BeF_3^- and Tyr97 are shown as sticks, Mg^{2+} is shown as sphere (red).

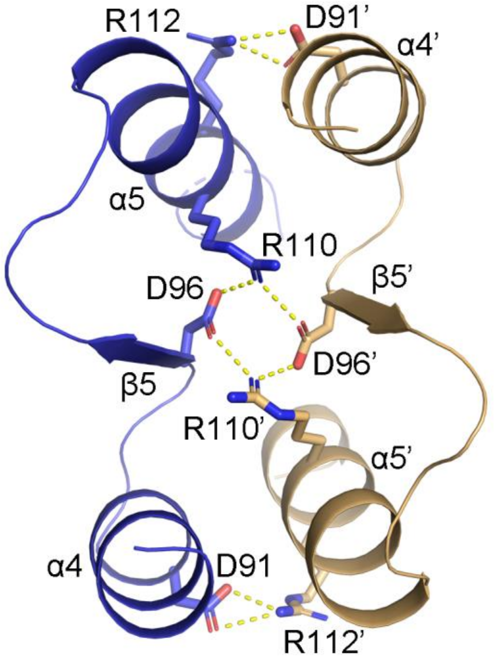

Supplementary Figure S6. The RD homodimer interface mediated by α4-β5-α5 secondary structure elements The conserved residues involving in generation of salt bridges between Asp96-Arg110 pair at the center and Asp91-Arg112 at out sides

of the interface are shown as sticks.

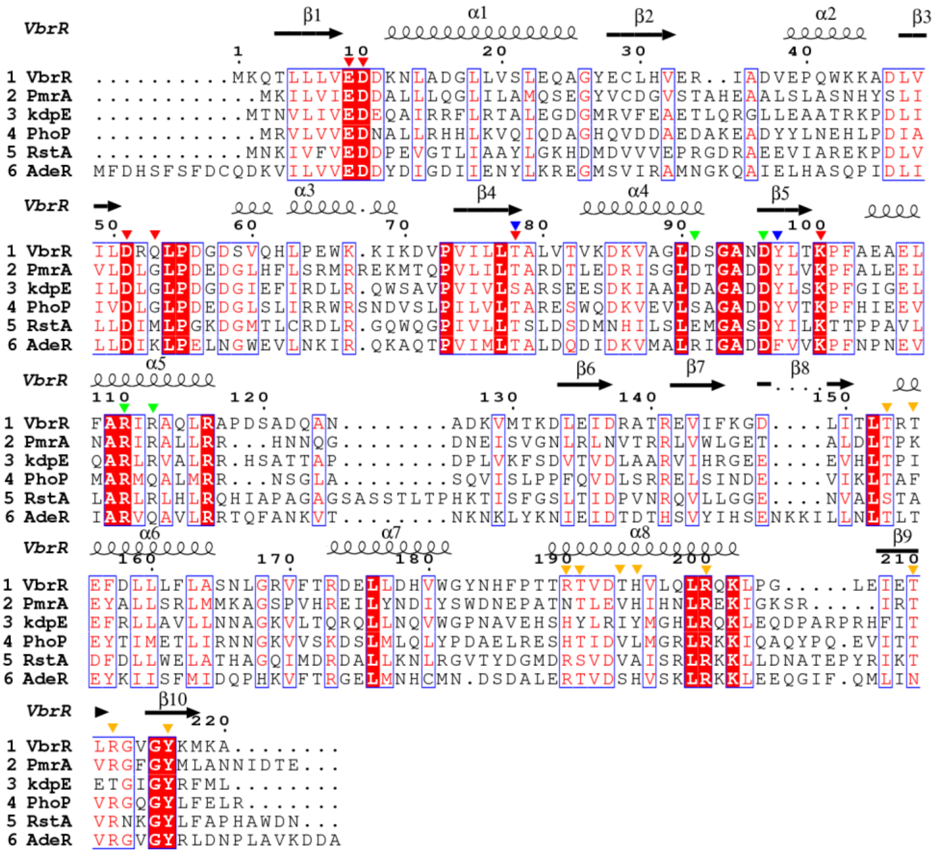

Supplementary Figure S7. Protein sequence alignment of the OmpR/PhoB subfamily response regulators They are *Vibrio parahaemolyticus* VbrR (VbrR), *Klebsiella pneumoniae* PmrA (PmrA), *Escherichia coli* KdpE (KdpE), *Mycobacteria tuberculosis* PhoP (PhoP), *K.pneumoniae* RstA (RstA), and *Acinetobacter baumannii* AdeR (AdeR). Secondary structure of VbrR are shown on the top. Residues interacting with BeF_3^- and Mg^{2+} are indicated with red triangles. Residues involving in generation of salt bridges of the RD homodimer are indicated with green triangles. Switch residues are indicated with blue triangles. Mutation sites on DBD involving the interaction with DNA are indicated with orange triangles.

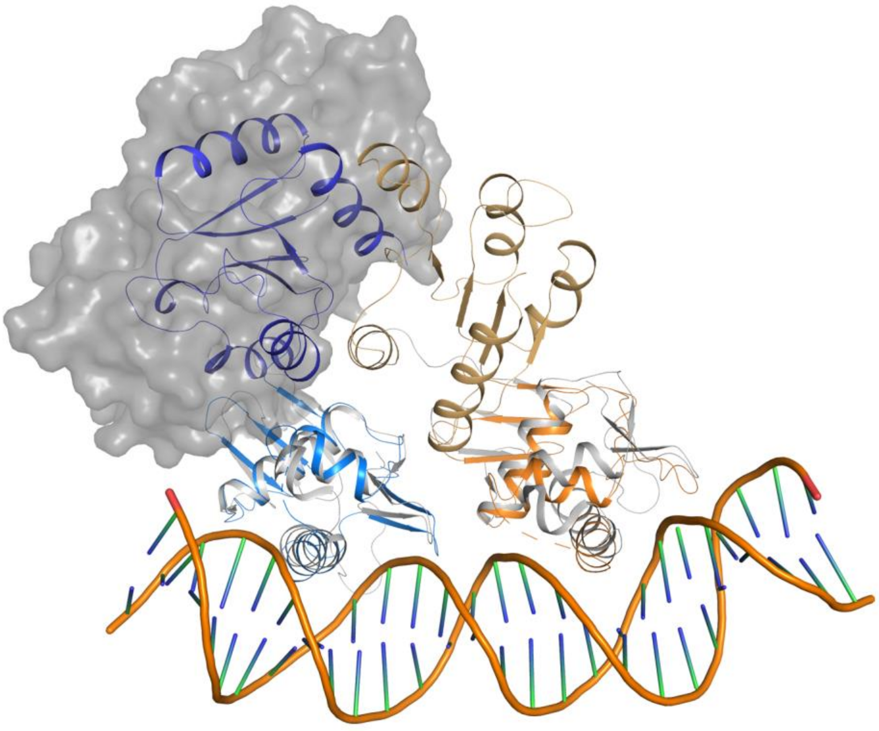

Supplementary Figure S8. Structural alignment of the VbrR-DNA and KdpE-DNA complexe The colors used for the VbrR-DNA complex are the same as those described in Figure 2A. The KdpE-DNA complex is colored gray. For clarity, the DNA of KdpE-DNA is omitted and the RD dimer of KdpE-DNA complex is shown as surface.

Supplementary Table S1. Data collection and model refinement statistics

	VbrR-DNA 7E1B	RD_BeF$_3^-$ 7E1H	Native-DBD 7E1F	SeMet-DBD 7E1D
**Data collection**				
Wavelength (Å)	0.97818	0.97891	0.97890	0.97852
Space group	C2	P65	$P2_12_12_1$	$P2_12_12_1$
Cell dimensions				
a (Å)	329.942	130.858	35.455	35.814
b (Å)	114.064	130.858	52.135	52.762
c (Å)	114.064	271.355	96.961	97.397
α , β , γ (°)	90,110.015,90	90,90,120	90, 90, 90	90, 90, 90
Number of molecules in ASU	8	14	2	2
Resolution (Å)	50.00-4.60 (4.76-4.60) [a]	29.47-2.81 (2.91-2.81)	30.00-1.45 (1.5-1.45)	30.00-2.00 (2.07-2.00)
Unique reflections	15994	59254	29399	12504
Redundancy	3.2	5.2	12.2	11.0
I/sigma (I)	14.9(0.86)	10.5(1.75)	37.34(4)	26.67(3.14)
R_{merge}	0.065(1.024)	0.174(0.904)	0.060(0.489)	0.109(0.570)
Completeness (%)	93.7(72.7)	99.9(100)	97.9(92.4)	99.5(96.3)
**Refinement**				
R_{work}/R_{free} [b]	0.265(0.321)	0.226(0.253)	0.1899/0.2163	0.1685/0.2121
Number of atoms	15460	15650	1553	1553
Protein residues	1668	1616	190	190
DNA	2132			
r.m.s.d.				
Bond lengths (Å)	0.005	0.005	0.005	0.008
Bond angles (°)	0.87	1.14	0.890	0.856
Ramachandran				
Favored (%)	92.3	92.9	95.7	94.1
Allowed (%)	7.5	6.9	4.3	5.9
Average B factor	140.32	38.01	20.51	28.93
Macromolecules	140.32	37.56	18.95	28.48
Ligand/Ion		23.87		
Water		39.97	30.79	34.42

a Numbers in parentheses are for highest-resolution shell

b $R=\Sigma_{hkl}||F_{obs}|-|F_{cal}||/\Sigma_{hkl}|F_{obs}|$, where F_{obs} and F_{calc} are observed and calculated structure-factor amplitudes, respectively. R_{free} was calculated as for R but using a subset (10%) of reflections that

were not used for refinement.

Supplementary Movie S1. VbrR precipitated quickly when BeF_3^- was added to the protein sample

Supplementary Movie S2. Conformational changes of DBD induced by phosphorylation
